# Microalgae Nutraceuticals

**DOI:** 10.3390/foods5030054

**Published:** 2016-08-22

**Authors:** Marcello Nicoletti

**Affiliations:** Department of Environmental Biology, University Sapienza of Rome, P.le A. Moro, 500185 Rome, Italy; marcello.nicoletti@uniroma1.it; Tel.: +39-0649-912-195

**Keywords:** microalgae, Spirulina, Chlorella, Klamath, food supplement, quality control

## Abstract

Among the new entries in the food supplements sector, an important place must be assigned to nutraceuticals containing microalgae, nowadays accounting for a large and rapidly expanding market. The marketed products are mainly based on three production strains, i.e., Spirulina and Chlorella, followed at a distance by Klamath. It is a composite situation, since two of them are cyanobacteria and the second one is eukaryotic. The reality is that each presents similarities in shape and appearance concerning the marketed form and several utilizations, and peculiarities that need special attention and adequate studies. First, general information is reported about the current scientific knowledge on each microalga, in particular the nutritional value and properties in prevention and wellbeing. Second, original studies are presented concerning the quality control of marketed products. Quality control is a key argument in nutraceuticals validation. Microalgae are particular organisms that need specific approaches to confirm identity and validate properties. The proposed control of quality is based on microscopic analysis of the morphologic characteristics. The final parts of this paper are dedicated to the need for specificity in uses and claims and to considerations about the future of microalgae in food supplements.

## 1. Introduction

International agencies like the FAO have announced the goal of food to feed everyone in the world [1,2]. However, to gain this success against any Malthusian prophecy, we must consider two main aspects, production and quality. Besides the eternal challenge concerning the production of a necessary quantity of food, recently quality is becoming fundamental for health maintaining and lifestyle improvements, as evidenced by the global obesity phenomenon [3,4,5]. Both aspects can be considered as decisive characteristics of the so-called nutritional environment. The nutritional environment is actually at the center of everybody’s attention, from governments to ordinary people, in consideration of the related problems including health and social costs.

Recently, rapid changes have impacted the food scenario, involving the appearance of several new products. These products—nutraceuticals, botanicals, and others—deeply influenced the market for their hybrid nature, located somewhere between ordinary food and medical drugs [6,7]. Actually, they are considered part of the food supplements sector, although the situation is continuously subject to changes and new interpretations [8,9,10]. The result is a complex, dynamic situation, needing a careful study and information about each of the different aspects [11].

A clear example of the actual and future situation of food, including food supplements, is offered by microalgae [12,13,14,15], nowadays accounting for a large and rapidly expanding market [16,17,18]. As a matter of fact, microalgae chemical composition is a complex mixture of minerals, vitamins, and primary and secondary products, offering a large spectrum of possible applications and utilizations for humans, from nutritional properties to antioxidant and anti-aging, also considering the preventative effects. In other words, microalgae are a case study in nutraceuticals.

On this occasion, after a set of necessary information on the raw materials, three arguments concerning microalgae products will be considered in detail:

(a) Quality control, a problem involving the whole food supplements market.
(b) The specificity of use and claim, since the derived products in food supplements are still not sufficiently tailored in terms of possible utilization.
(c) The real need of microalgae for mankind, in particular as nutraceuticals.

## 2. The Evolution of Food Supplements

**Figure 1:**
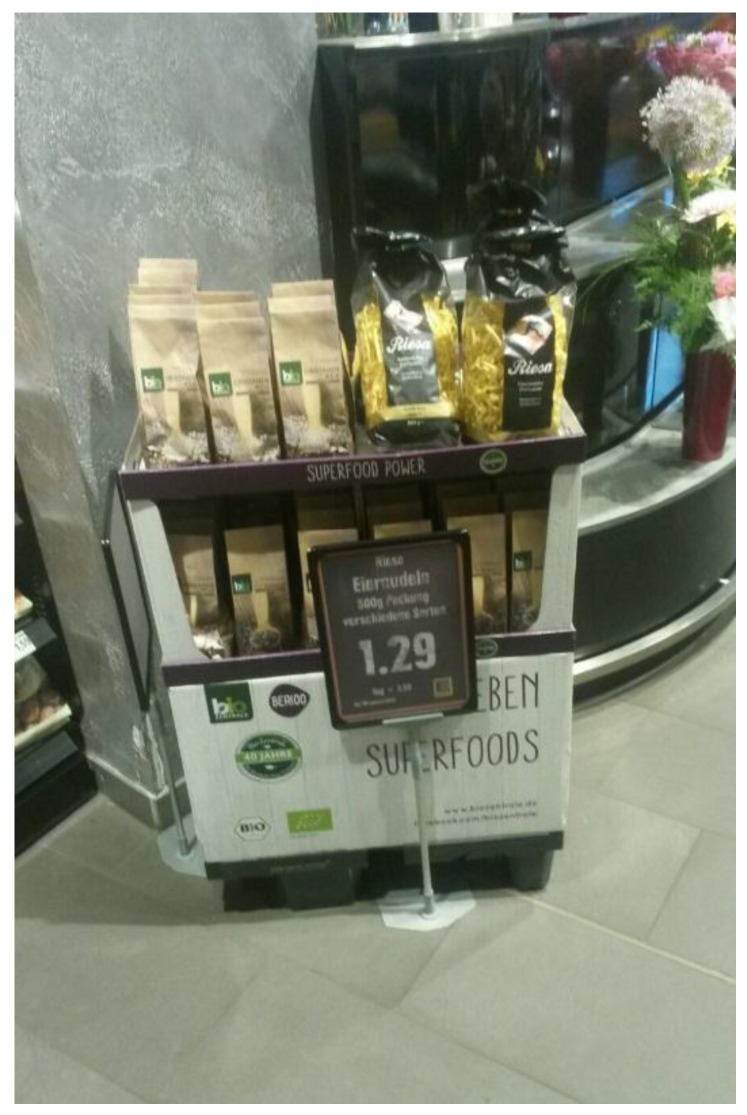
So-called superfoods can now be easily found in supermarkets.

At the beginning of their appearance in the market, food supplements were considered as concentrated nutrients useful to support nutritional needs and/or supply alimentary deficiencies in the ordinary diet and, consequently, their composition clearly evidenced the presence of vitamins, minerals, proteins, and carbohydrates [8]. Later, their composition showed a massive introduction of “other substances,” mainly consisting of extracts of medicinal plants. Composition, targets, and marketing clearly changed, as well as denominations, with possible names including nutraceuticals (the most frequently used, but still not officially recognized), dietary supplements, medical devices, herbal drug preparations, traditional medicine herbal products, botanical food supplements, botanical supplements, or simply botanicals, in the case of utilization of raw plant materials [10,11]. The main difference consists in the organic composition, since food supplements of the first generation mainly contain substances of primary metabolism, like carbohydrates, vitamins, and proteins, whereas other substances, typical of nutraceuticals, are secondary products, like flavonoids, terpenes, polyphenols, organic acids, pigments, etc., usually present in raw plant materials or extracts. This simplified scheme is contradicted by microalgae, which are a rich source of both types of substances, as evidenced by the reported chemical analyses. Similar to food supplements evolution, for a long time seaweed was mainly considered a source of animal feed and human alimentation, but the introduction in the market of nutraceuticals radically changed the scenario. To deal with this particular situation, the attractive term “superfood” was suggested [19]. The so-called superfoods are now present in supermarkets and herbal shops, although their composition and nature were not determined and clarified (Figure 1).

The third type of food supplements, now emerging, is functional foods or pharmafoods, based on the addition of special ingredients with certain physiological properties to ordinary foods. This is probably the best future use of microalgae, bypassing the limits of attractiveness in the current utilizations and opening the way for a wide variety of different products, like green pasta or special desserts.

## 3. Aquatic Autotrophics

Life depends on water. Seaweed, together with animals and plants, is fundamental to the current food scenario [20,21]. Ordinary people may not aware, but a standard diet is full of seaweed products, directly or indirectly, and not even considering that planet life largely depends on the photosynthetic work of phytoplankton and that most living organisms are marine. The main biomass of seaweeds present in the planet are microscopic unicellular organisms named microalgae. Microalgae are not only naturally abundant in the sea and terrestrial water, but can be cultivated easily and in large quantities, giving rise to a low-cost raw material with many potential uses. Food supplements were able to enlarge the application horizon of microalgae, but this is probably only the beginning.

For a long time, seaweed use, including microalgae, was limited to livestock feed and fertilizer. Recently, new important uses for microalgae have been emerging, fuelled by increasing interest and curiosity from consumers. Again, technology has a fundamental role in transforming raw materials into a myriad of different products. From food to biodiesel, the microalgae empire is coming [22,23,24,25,26,27].

Algae are a polyphyletic group of autotrophic marine organisms (alga in Latin means marine plant), erroneously considered as plants due to the common photosynthetic trophic pathway. Generally speaking, they are known as seaweed; however, “seaweed” is a colloquial term and lacks a formal definition. In addition, some tuft-forming blue-green algae are sometimes considered to be algae, but they must be linked to prokaryotes (cyanobacteria) [28,29]. The classic classification of algae, now obsolete, is based on colour, being the most direct evident morphologic character. Colourations derive from different pigments associated with the photosynthetic process, according to the water depth where they live. Therefore, we have blue-green, yellow, green, brown, and red algae. Algae can be unicellular or multicellular, microscopic or giant, simple or complex, similar in form to plants or to bacteria. Inside these organisms, we can read the complete story of autotrophic living beings, from the beginning until 450 million years ago, when life left the water. Primordial microalgae were very similar to those present in our seas. Although primordial habitats were totally different from current ones, these cells have been able to survive practically unchanged until now and are still the main part of the biomass of the planet. Considering their massive distribution, they are still dominating the planet.

The potential use of microalgae is enormous: three-quarters of the planet’s surface is occupied by water and most marine water is available for life. The space habitable by marine organisms is far greater to that available for terrestrial plants. Microalgae, which cover almost 75% of algae species, contribute approximately 40% of the oxygen in the atmosphere. Despite the apparent simplicity of their cells, at least 40,000 species of microalgae phytoplankton have been identified [29]. The key of this success is in the metabolism, i.e., in the completeness of substances present in the cytoplasm. This is the key to their importance in nutraceuticals. For this reason, in this paper the analytical part is a particular focus.

## 4. Cyanobacteria in Nutraceuticals

The market for microalgae nutraceuticals is dominated by two cyanobacteria, universally known as Spirulina and Klamath, and the chloroficean Chlorella. To understand cyanobacteria, we must start with the outset of life, 4.5 billion years ago, when these microscopic cells started the experiment of life [20]. From that beginning, organic life was separated by the trophic level. On one side were the autotrophic cyanobacteria, and on the other side were the heterotrophic consumers, the Eubacteria, which use the organic substances produced by the former. Besides them, Archaea prokaryotes were experimenting with other methods of producing energy and organic matter. We are here because that model, despite the apparent simplicity, was (and still is) successful; its capacity is nowadays testified to by its presence. Despite the absence of a nucleus, bacteria possess all the biochemical tools to produce any kind of products, including precious essential amino acids and enzyme supply [30,31,32].

Cyanobacteria are also known as blue-green algae and are traditionally associated with seaweed, in consideration of the trophic level, the environmental condition, and several other similarities. The evolutionary line leading to modern plants started from that model based on the chlorophyll work in autotrophic algae. Following the evolutionary pathways, we focused on advanced organisms, considering them a major source of food and medicinal drugs. It is now time to reverse this attitude. Again, the first signal of novelty comes from nutraceuticals. Microalgae are heavily used as raw materials in food supplements. The claim is to obtain both equilibrium in the diet and a specific activity, in accordance with the characters of the functional food.

Production of microalgae must be carefully considered. Microalgae need some special conditions to produce large quantities of biomass. This can be obtained in natural, as well as artificial, habitats. Attention must be focused on algal bloom, which in some periods can interest particular species in certain seas, as the result of optimal biological, physical, and chemical conditions. The resulting toxic water contamination can be dangerous directly or in the food chain [33]. On the other side, certain species of microalgae can be successfully used to clean contaminated gas or water, in particular from industrial production [23].

## 5. Spiralated Cianobacteria

### 5.1. Spirulina

**Figure 2:**
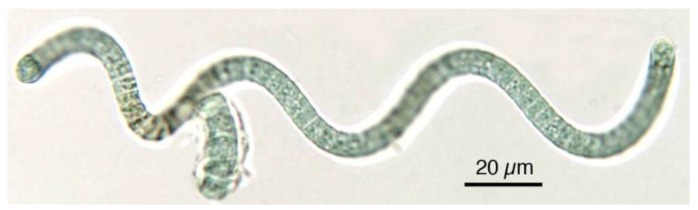
In Spirulina, cells are aggregated into filaments that tend to form spirals. Note the blue-green colour typical of cyanobacteria.

**Figure 3:**
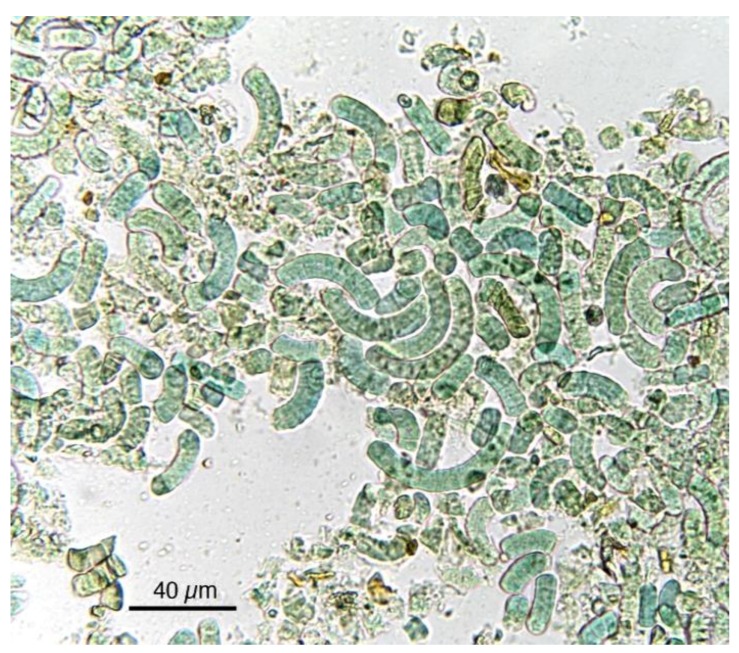
Most Spirulina cells, after their exsiccation and transformation into marketed products, are fragmented, but the original characteristics useful for identification are still evident.

Spirulina (classified as *Arthrospira* sp*.*) is a cyanobacterium present in free-floating filaments in the form of an open left-hand helix characterized by cylindrical multicellular trichromes (Figure 2 and Figure 3). It occurs naturally in tropical and subtropical alkaline hot lakes with high pH values and high salt concentrations, like carbonate and bicarbonate. Two species, *S. platensis* and *S. maxima*, are mainly present, the first occurring in Africa, Asia, and South America, whereas the second is confined to Central America. Cultivation of Spirulina on a large scale started 30 years ago in Mexico and China, and later in other parts of the world, owing to the easy conditions for cultivation. Most cultivated Spirulina is produced in open channel raceway ponds, with paddle-wheels used to agitate the water. The largest commercial producers of Spirulina are located in the USA, Thailand, India, Taiwan, China, Bangladesh, Pakistan, Burma (Myanmar), Greece, and Chile. Spirulina is primarily known across the world for its potential nutritional value. It is one of the rare edible bacteria, due to its low purine concentration, which allows it to pose a minimal risk of uric acid build-up in the body [22]. The food industry classifies *A. platensis* as a single-celled protein, meaning that it is an edible microbe with a high food value [32]. The nutritional value of Spirulina was already known to the Aztecs, who harvested the alga from Texcoco Lake, near Mexico City. Spanish soldiers led by Cortes described its use as a daily food and the sale as cakes [33,34]. It is rich in vitamins, minerals, β-carotene, essential fatty acids, and antioxidants, all of which have facilitated its commercial production as a human food supplement over the course of the past decade [35,36]. Its consumption has been shown to have cardiovascular positive effects, lowering blood pressure and reducing cholesterol [36]. In consideration of its anti-carcinogenic properties, it was used to treat radiation sickness in people that were affected by the 1986 Chernobyl nuclear accident [37,38,39,40].

Nowadays, it is used in food supplements, in the form of tablets or power, alone or in association with other algae or plant extracts, for human or animal uses. Actually, Spirulina is considered a good source of vitamins and essential amino acids. It also has very high protein content with a well-balanced composition, making it even more desirable as a food supplement [31,32]. It is also noteworthy for its oil content, in quantity (7%) and in quality (α-linolenic acid (ALA), linoleic acid (LA), stearidonic acid (SDA), eicosapentaenoic acid (EPA), docosahexaenoic acid (DHA), and arachidonic acid (AA)). The content in vitamins, like those of the group B, and hydrocarbons is considered relevant and complete [31].

## 6. Algae in Environmental Niches

### 6.1. Alga Klamath

**Figure 4:**
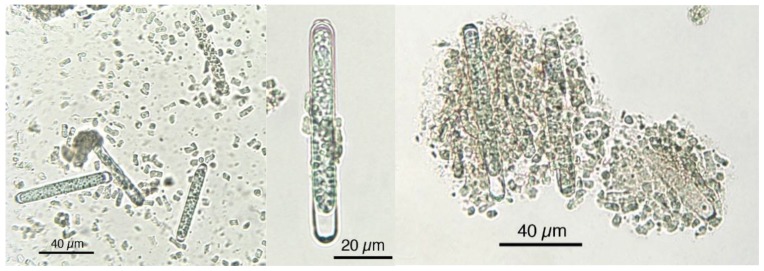
Microphotographs of Klamath power showing fragmented as well as intact cells with resistant cell wall and characteristic form.

Klamath is the name of a lake in Oregon (USA, perimeter 161 km, medium deep 2.4 m), where the cyanobacterium *Aphanizomenon flos-aquae* grows spontaneously and therefore is commonly known as alga Klamath (Figure 4). Several other *Aphanizomenon* spp. occur in other parts of the planet, but Lake Klamath allows for good production and easy harvesting. It can be considered a living fossil, survived into a particular ecological niche [41]. Therefore, alga Klamath’s environmental condition is very different from that of Spirulina. Spirulina algae can be grown in controlled conditions that do not exclude completely contaminations, but Klamath microalgae are in a natural and very large place, open to the presence of other microalgae and microorganisms and subjected to seasonal cycles. In March, the *Fragilaria* spp. dominate the phytoplankton of the lake, followed by *A. flos-aquae* between May and July. In the same period, also *Anabaena flos-aquae* (less that 1% of the total algae) and later in July–October *Microcystis aeruginosa* and *Coelopshaerium* are present. Therefore, during the collection of Spirulina, several algal species can be collected, giving rise to warnings about the presence of neurotoxins produced by other algae, in particular about *Anabaena*, *Microcystis*, and *Oscillatoria* spp. that are normally present in the lake and can produce toxins. However, so far there is not complete and reliable information about the production of toxins, like microcystins, from *A. flos aque*; analyses on Lake Klamath phytotoxins, as well as on marketed products, are a controversial matter, including the analytic method, although all reports only concern the possibility of contamination and toxic effects [42].

Both Spirulina and alga Klamath are marketed with suggestive adjectives, such as *superfood* or *the food of the future*. Several activities are reported and in part confirmed by different types of experiments and clinical trials; however, as for several food supplements, the scientific validation is not considered complete. In the first place, despite the poorly inviting taste, the nutritional value and the anti-obesity effect are the object of considerable marketing appeals.

### 6.2. Chlorella

**Figure 5:**
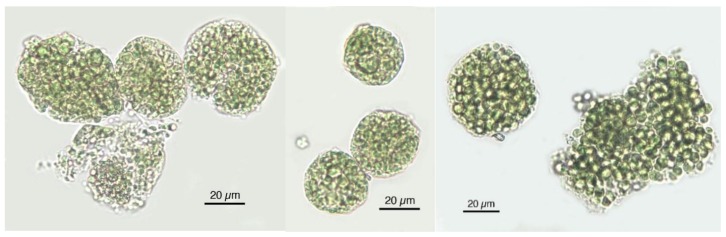
The microscopic unicellular Chlorella cells tend to agglomerate also in food supplements.

Chlorella is a microscopic unicellular seaweed pertaining to the green algae (Chlorophyta) (Figure 5). The evident green pigmentation is due to the presence of the two chlorophylls, *a* and *b*, the same ones in terrestrial plants. It can be easily cultivated in simple conditions, producing enormous quantities of biomass in little time. It needs only water, CO_2_, light, and a small quantity of minerals. Considering its quantity of proteins, amino acids, minerals, vitamins, and pigments [43,44], it should be considered the ideal food [45].

**Figure 6:**
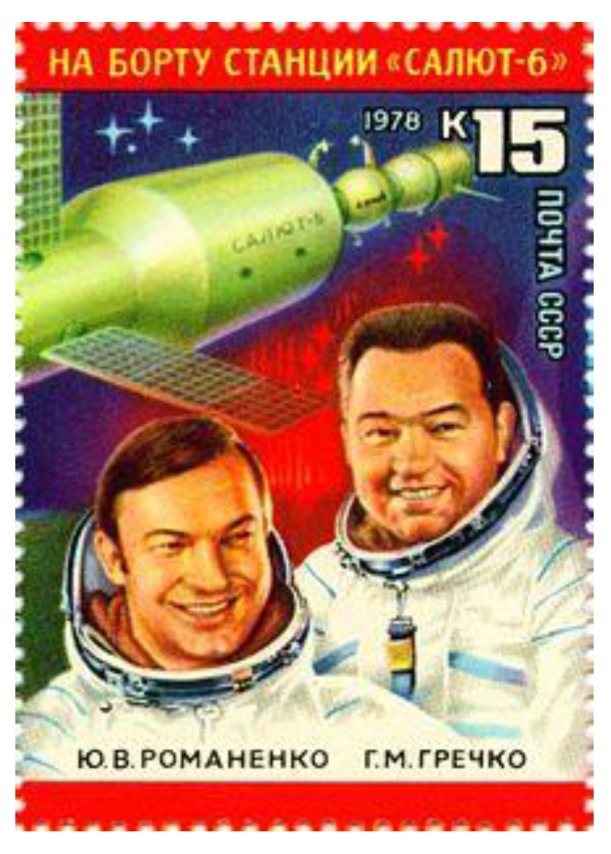
The soviet cosmonaut Yury Viktorovich Romanenko tested the utilization of Chlorella as an ideal food in long space journeys.

Its properties and capacities have been idealized and emphasized in various ways. Yury Viktorovich Romanenko, cosmonaut, twice hero of the Soviet Union, holds the record for the longest stay in space, with a total of 430 days, 20 h, 21 min, 30 s in several missions, before the space station came about (Figure 6). During his time in space, he was able to perform a series of important experiments on seaweed of the *Chlorella* genus, in consideration of its future utilization as a food in long space journeys. However, simple chemistry is not the only consideration. The flavour of this seaweed, as well as of other ones, is not enjoyable in comparison with ordinary dishes. Also, in this case, there is a long list of assigned health activities and properties, including the detoxicant action and the stimulation of the immune system. On the side of wellbeing, evidence of improved digestion and normalization of sugar metabolism has been reported.

## 7. Quality Control in Microalgae

There is a concern about toxins produced by cyanobacteria [33]. Microcystins, produced by *Microcystis* sp., were in the news as they caused acute liver failure in more than 100 Brazilian haemodialysis patients. The problem arose due to the use of a contaminated water reservoir, whose filters and carbon adsorption tanks had not been changed for a long time. Another typical alarm comes from the consumption of saltwater mussels (*Mytilus edulis*), which feed on *Microcystis* and accumulate microcystins persisting for several days after the transfer of the mussels into clean water [46]. In Southern Italy, there is a tradition of consumption of fresh mussels, without any form of cooking, causing in some cases acute gastrointestinal problems and dysentery, albeit in some cases it is unclear that the problems were attributable to the mussels. When production is operated in clean water and controls are performed, no alarms or problems were registered. Another case comes from another species, *Pfiesteria* sp., which produces neurotoxins. In 1997 in Maryland, USA, the so-called “*Pfisteria* panic” produced a 210% reduction in sales of fish and shellfish due to a public perception of danger [47,48]. Harmful seaweed blooms are caused by the unusual proliferation of certain toxic microalgae, which are regular constituents of the plankton microflora found in the Austral ecosystem of Chile, when environmental conditions are favourable to their blooming [49,50,51]. They can be due to the presence of toxins, as occurs with the dinoflagellates responsible for paralytic shellfish poison and diarrhoeic shellfish poison. Therefore, some of the marine organisms that filter microalgae, such as bivalve shellfish, concentrate these toxins. Consumption of these organisms may seriously harm human health and may even be lethal.

In food supplements, only the reported microalgae species are utilized and they are considered devoid of any toxin production. They are used alone or together. The marketed raw material consists of lyophilized or dried seaweed as a fine green powder, so that is very difficult to ascertain at a glance the identity of the utilised species. A control is necessary, not least because the cost of each one is very different and therefore the combination is not always reported on the label, but the real danger comes from the incidental occurrence of toxic algae.

**Figure 7:**
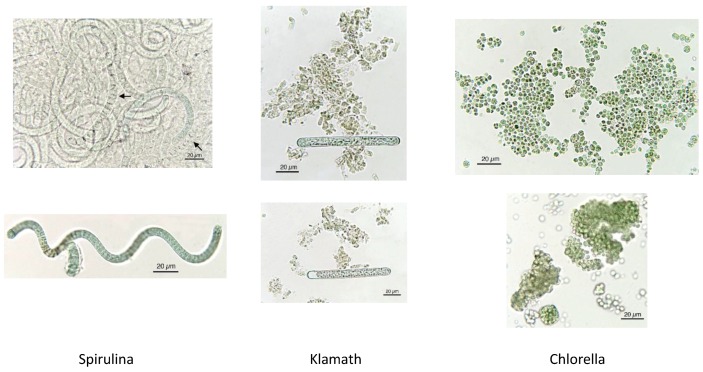
Microscopic characteristics of each microalga, allowing for identification.

**Figure 8:**
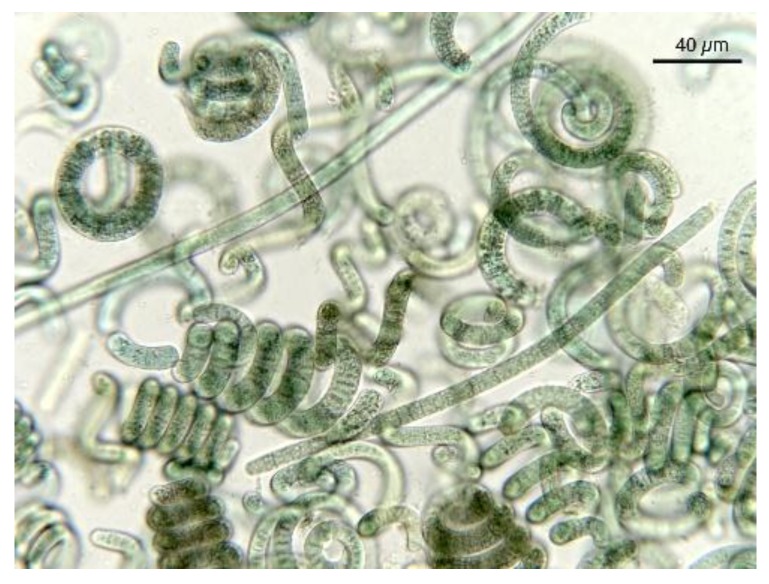
Spirulina product showing the presence of *Oscillatoria* sp. (long linear cells).

**Figure 9:**
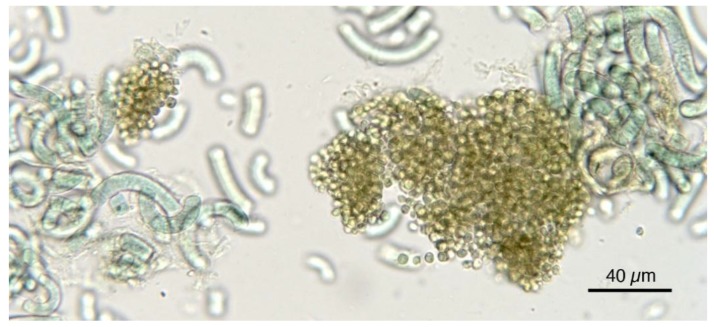
Spirulina product showing the presence of *Chlorella*-like and/or unicellular cyanobacterial cells.

As evidenced in Figure 7, each microalga utilized in nutraceuticals possesses a distinct microscopic morphologic shape. The morphological analysis at the microscope is very useful and the results prove the presence of alien species. Figure 8 shows the presence of *Oscillatoria* sp. in a Spirulina commercial sample, whereas in Figure 9 the co-occurrence of Spirulina and Chlorella is shown. However, to obtain a reliable result, the quality control needs a specialized treatment, a good instrument, and a specialist in algology able to identify the species, in particular in case of the presence of alien toxic microalgae that can be easily cultivated, or casually present, together with the required ones. Furthermore, during the process of preparation of tablets, the delicate original form of the microalgae can be partially destroyed. In particular, high-quality images should be obtained and presented, as those reported here. Also, a sufficient number of analyses is necessary to ascertain the amount of contamination. In case of contamination, it is necessary to identify the alien species, but the presence of a contaminant species, like in Figure 8, must be considered only as an indication of the possible presence of toxins in the marketed products.

## 8. The Importance of Microalgae in Food Supplements

Nowadays, microalgae are mainly utilized to feed livestock and pets. However, human consumption is increasing, in particular in food supplements. There are several cases of interest in the use of microalgae in food supplements. Vegans adopt a diet characterized by the practice of abstaining from the use of animal products [52]. Generally, the diet is associated with a philosophy that rejects the commodity status of animals and other ethical tendencies [53]. Therefore, vegans and omnivores can get into confrontations and debates [52,54]. So far, vegans seem to be victorious, with their numbers constantly increasing. Vegans account for ca. 5% of the total population in Israel, 2% in the United Kingdom and United States, and 1% in Germany and in Italy, which means anywhere from several thousand to seven million inhabitants.

The vegan diet is often considered nutrient-deficient, due to unbalanced protein sources and a low intake of some vitamins and minerals. Recently, some scientific data has been produced that gives substance to the debates. A recent study reports the results of a comparison between a group of vegetarians and a group of non-vegetarians for an average period of eight years [55]. The study stated: “A vegan diet should involve a balanced intake of whole grain products, legumes, seeds and sources of proteins, as well as vegetables, fruits, berries and unsaturated fats. In addition, vegans should consume calcium-fortified drinks and use vitamin B12, vitamin D and iodine supplements to complete their diet.” The results mean that vegans face nutritional problems due to a shortage of vitamin B12, 25-hydroxyvitamin D, selenium, and long-chain omega-3 fatty acids.

Factors that influence vegetarian food intake should include knowledge of a balanced diet, vegetarian food variety, as well as the use of enriched food items and food supplements. Among the available food supplements, for their content microalgae seem one of the best candidates to supplement vegans’ and vegetarians’ diet.

The vegan diet debate, including the recent WHO alert concerning the need for limiting meat intake, evidences the tendency to obtain health implements by a hypocaloric intake. This aspect has been recently considered in a study reported by an interdisciplinary group [56,57,58,59,60]. The paper, published in *Cell Metabolism* in 2015, showed that in mice a diet alternating prolonged fasting (PF) and a nutrient-rich medium resulted in extended yeast lifespan (+20%), independently of established pro-longevity genes [60]. Besides extended middle-age longevity, bi-monthly FMD cycles of four days of a diet that mimics fasting (FMD), developed to minimize the burden of PF, lowered visceral fat, reduced cancer incidence and skin lesions, rejuvenated the immune system, and retarded bone mineral density loss. In old mice, FMD cycles promoted hippocampal neurogenesis, lowered IGF-1 levels and PKA activity, elevated NeuroD1, and improved cognitive performance. In a pilot clinical trial, three FMD cycles decreased risk factors/biomarkers for aging, diabetes, cardiovascular disease, and cancer without major adverse effects, providing support for the use of FMDs to promote health span. The results and conclusions of the research find an independent confirmation in Laron Syndrome. The Laron population lives in remote villages of Ecuador. They are very small people, suffering from an incredibly rare genetic disorder that stops them from growing taller than 4 feet and produces elderly features even in children. The syndrome is a consequence of low IGF-1 levels, but the same factor also seems to protect them against cancer and diabetes, and maybe even heart disease and Alzheimer’s.

Therefore, the mima hypocaloric diet shows that: (a) a high protein intake is linked to increased cancer, diabetes, and overall mortality; (b) high IGF-1 levels increase the relationship between mortality and high protein levels; (c) higher protein consumption may be protective for older adults; and (d) plant-derived proteins are associated with lower mortality than animal-derived proteins. Therefore, in this case also, microalgae can play a role in the diet.

## 9. Conclusions

As already reported, algae food supplements can be particularly useful to support some diets. However, some aspects need to be considered and research should play a central role. Microalgae are considered more or less at the same level of utilization in nutraceuticals, albeit with several differences. Dried spirulina used in food supplements contains about 60% (51%–71%) protein, with a composition rich in all essential amino acids, though with reduced amounts of methionine, cysteine, and lysine when compared to meat, eggs, and milk, although superior to typical plant protein, such as that from legumes [32,61,62,63]. Furthermore, an interesting debate concerns vitamin B12. Most edible cyanobacteria, like spirulina, do not naturally contain vitamin B12, but predominantly contain pseudovitamin B12, which is inactive in humans [64,65]. Therefore, the American Dietetic Association and Dieticians of Canada, in their position paper on vegetarian diets, state that spirulina cannot be counted on as a reliable source of active vitamin B12 [66]. However, companies that grow and market spirulina have claimed it to be a significant source of the vitamin on the basis of alternative, unpublished assays, although their claims are not accepted by independent scientific organizations. However, there is a general rule to be considered: plant drugs contain plenty of secondary metabolites acting as non-active biochemical precursors, usually named pro-drugs, from alliin in garlic to THC-A in cannabis, as reported in Table 1. There are several reported reasons for its situation: active products are precious and must be preserved or the metabolite must act on the right please, etc.

The vitamin B12 debate is only an example of the general debate concerning the real necessity and effectiveness of nutraceuticals consumption. Probably, more research is needed on these as on many other food supplements, in order to obtain the right utilization. Food supplements, so far fuelled by a billion-dollar market, urgently need scientific validation.

## Figures and Tables

**Table 1:** Examples of plant pro-drugs.

Prodrug	Active Substances
Glucosinolates	Isothiocyanates
Alliin	Allicin, ajoenes
Cumaroylglucoside	Coumarin
Arbutin	Hydroquinone
Salicin	Saligenin, salicylic acid
Bi-desmosidic saponin	Mono-desmosidic saponins
Ranunculin	Protoanemonin
THC-A	THC
Proto-vitamin B12	Vitamin B12
Cyanogenic glucoside	HCN
Rhein, sennosides	Antraquinonic aglucone
Hennosides	Lawsone
Vanilloside	Vanillin
Gein	Eugenol
Methylazoxymethanol	Cycasin
